# Hypercoagulability as a prognostic factor for survival in patients with metastatic renal cell carcinoma

**DOI:** 10.1186/1756-9966-28-30

**Published:** 2009-03-02

**Authors:** Ilya V Tsimafeyeu, Lev V Demidov, Albina V Madzhuga, Oksana V Somonova, Anna L Yelizarova

**Affiliations:** 1 Department of Biotherapy, Clinical Research Laboratory, N.N. Blokhin Russian Cancer Research Center, Kidney Cancer Research Bureau, Moscow, Russia

## Abstract

**Background:**

In experimental systems, interference with coagulation can affect tumor biology. We suggested that abnormal coagulation could be a negative predictor for response to immunotherapy and survival among patients with metastatic renal cell carcinoma (MRCC).

**Methods:**

To address this issue, retrospective analysis of 289 previously untreated MRCC patients entering on institutional review board-approved clinical trials was conducted between 2003 and 2006. In addition, two groups of MRCC patients with (n = 28) or without (n = 28) hypercoagulability were compared in a case-control study. Baseline and treatment characteristics were well balanced.

**Results:**

Hypercoagulability was present at treatment start in 40% of patients. Median baseline fibrinogen was 6.2 mg/dl. Serious disorders were found in 68% of patients. Abnormal coagulation was strongly associated with a number of metastatic sites (2 and more metastatic sites vs. 0–1 (P = .001). Patients with high extent of hypercoagulability had significantly higher number of metastatic sites (P = .02). On univariate analysis, patients with hypercoagulability had significantly shorter overall survival than patients with normal coagulation; median survivals of 8.9 and 16.3, respectively (P = .001).

Short survival and low response rate also were significantly associated with hypercoagulability in a case-control study. Median survival was 8.2 months and 14.6 months, respectively (P = .0011). Disease control rate (overall response + stable disease) was significantly higher in patients with normal coagulation: 71.4 versus 42.9% (P = .003).

**Conclusion:**

Hypercoagulability disorders were found to be prognostic factor for response rate to systemic therapy and survival in patients with MRCC.

## Background

Renal cell carcinoma (RCC) is the most common cancer of the kidney [1]. An estimated 16,000 new cases of RCC were diagnosed in Russian Federation in 2005. Up to 30% of patients with RCC present with metastatic disease every year, and recurrence develops in approximately 40% of patients treated for localized tumor [2].

High-dose interleukin-2 therapy rarely induces a durable complete response, and interferon alpha provides only a modest survival advantage. Overall response rate with these cytokines is low (5 to 20%) [3]. A growing understanding of the underlying biology of RCC has led to development of vascular endothelial growth factor (VEGF) inhibitors, such as sunitinib and sorafenib [4,5]. The promising data with VEGF inhibition in metastatic RCC have established new opportunities for improving outcomes in this historically resistant malignancy. Combination of targeted therapy and biological agents has promising results. However, several questions remain unanswered concerning their optimal use.

Improved treatment strategies and/or better methods of identifying those patients likely to benefit from medical therapy are needed.

Considerable data is now available to help predicting the outcome for patients with advanced renal cancer receiving systemic therapy. Factors that have been variably associated with response and survival include Karnofsky performance status < 80%, time from diagnosis to treatment < 12 months, corrected serum calcium > 10 mg/dL, Hemoglobin below the lower limit of normal, and LDH > 1.5 times the upper limit of normal. Patients considered to have a favorable profile are those with no poor prognostic factors present; intermediate group patients have 1–2 factors present; and patients with an unfavorable profile have > 2 factors present. This is a Memorial Sloan Kettering Cancer Center (MSKCC) model developed by Motzer et al. [6,7].

Several poor prognostic factors have been identified in ARCC trial (efficacy and safety of temsirolimus in previously untreated patients with metastatic RCC), such as number of organs with metastases (2 and more) and interval from original diagnosis to the start of systemic therapy [8].

Moreover, disorders in hemostatic system such as hypercoagulability can impact on tumor growth.

We evaluated rate of abnormal coagulation in metastatic RCC, correlation between levels of disorders, number of metastatic sites; determine response rate, disease progression and survival in patients with or without abnormal coagulation who had received immunotherapy.

## Methods

### Patients

The study population consisted of patients who had metastatic RCC with any type of histology. Patients who had not received previous systemic therapies for metastatic disease were included in the analysis. Other key eligibility criteria for analysis included the presence of measurable disease, adequate hepatic, renal, and cardiac function. Patients were ineligible if they had brain metastases, life expectancy of less than 4 month, thrombocytosis, indication for anticoagulant treatment (for example, mechanic heart valves, inferior vena cava filter, previous venous thromboembolism, or atrial fibrillation), medical contraception.

### Study design and methods of evaluation

Retrospective analysis of 289 patients entering on institutional review board-approved clinical trials was conducted between 2003 and 2006 at the N.N. Blokhin Russian Cancer Research Center.

In addition, two groups of patients with (n = 28) or without (n = 28) hypercoagulability were compared in a case-control study. Baseline and treatment characteristics were well balanced. All 56 patients previously received at least 2 cycles of low-dose immunotherapy (interleukin-2, 1 MU, i.v, 3 tiw and interferon alfa 2b, 5 MU, s.c, 3 tiw – 3 weeks on, 3 weeks off). Patients were compared by MSKCC prognostic score. We measured the primary end-point (differences in disease progression rate) from the date of treatment start until the date of evaluation for response rate after two treatment cycles, and the secondary end-point (median overall survival) from the date of treatment start until the date of death. The progression of disease was determined on the basis of findings of computed tomography (CT) or magnetic resonance imaging (MRI), clinical progression, or death, with the use of the Response Evaluation Criteria in Solid Tumors (RECIST).

Factors evaluated in all patients were: age, gender, time from diagnosis to on-study, number of metastatic sites, MSKCC prognostic factors, fibrinogen, fibrin monomer, and D-dimer. The coagulation profile was assessed before the start of the treatment. Pretreatment level was used to classify patients by the presence or absence of hypercoagulability. Hypercoagulability was defined as elevation of main coagulation factors (Table 1). Normal coagulogram was defined as normal values of fibrinogen (≤ 4.0 mg/dl), D-dimer (≤ 0.248 mg/ml) and negative fibrin monomer. Patients who initially had normal levels of coagulation factors and later developed hypercoagulability were categorized as having normal coagulation and were included in the analysis.

**Table 1:** Extent of hypercoagulability

**Extent of hypercoagulability**	**Fibrinogen, mg/dl**	**D-dimer, mg/ml**	**Fibrin Monomer**
**Low**	4.01–5	0.249–0.5	+

**Intermediate**	5,01–6	0.51–1	++

**High**	> 6.01	> 1.01	+++

All coagulograms were performed on an automatic STA COMPACT analyzing device.

### Statistical analysis

The hypercoagulability was summarized using frequency counts. Summary statistics (Mean, Median, and Proportion) was used to describe patient baseline characteristics. An estimate of the overall response rate/disease progression rate was made by taking number of patients with a response/progression of disease (number of evaluable patients). The secondary endpoint was a difference in overall survival between patients treated with immunotherapy and hypercoagulability versus patients with normal coagulation was tested using a 2-sided Log-rank test (α = 0.05). Patients alive at the end of follow-up were censored. The Kaplan-Meier method was used to estimate survival outcomes. Multiple factors were assessed using Cox proportional hazards regression model. The chi-square test and Fisher exact test were used to compare patient groups.

## Results

### Demographics

Two hundred and eighty nine untreated patients were enrolled on trials. Seventy-eight percent of patients were males, and median age was 61.8 years. The demographics are described in Table 2.

**Table 2:** Patient and disease characteristics

**Factor**	**No. (%)**	**% with hypercoagulability**	***P***
Hypercoagulability			
No	173 (60)	-	-
Yes	116 (40)	-	-

Extent of hypercoagulability			
Low	13 (11)	-	-
Intermediate	24 (21)	-	-
High	79 (68)	-	-

Age			
< 60	107 (37)	34	
≥ 60	182 (63)	44	.004

Gender			
Male	224 (78)	39	
Female	65 (22)	45	.61

ECOG			
0	110 (38)	38	
1	170 (59)	41	
2	9 (3)	44	.07

Prior nephrectomy			
No	25 (9)	48	
Yes	264 (91)	40	.03

Time from diagnosis to on-study			
≥ 1 y	165 (57)	30	
< 1 y	124 (43)	53	< .001

Number of metastatic sites			
0, 1	125 (43)	17	
≥ 2	164 (57)	58	.001

Bone metastasis			
No	199 (69)	38	
Yes	90 (31)	44	.06

Liver metastasis			
No	211 (73)	34	
Yes	78 (27)	56	.02

MSKCC prognostic groups			
Favorable	121 (42)	46	
Intermediate	64 (22)	22	
Poor	104 (36)	44	.84

Histology			
Clear cell	231 (80)	42	
Non-clear	58 (20)	33	.04

Venous thrombosis			
No	275	37	
Yes	14	100	-

Of 289 patients whose medical charts were reviewed, hypercoagulability was present at treatment entry in 40% of patients. Median baseline fibrinogen was 6.2 mg/dl (95% CI; 3.4–9). Thirteen (11%), 24 (21%), and 79 (68%) coagulation profiles were classified as low, intermediate, or high grade hypercoagulability based on the previously described model.

We analyzed association of hypercoagulability with MSKCC prognostic factors as well as number of metastatic sites. 46, 22 and 44% patients in groups of favorable, intermediate and poor prognosis respectively had hypercoagulability. Abnormal coagulation was strongly associated with number of metastatic sites (2 and more metastatic sites vs. 0–1 (P = .001). Patients with high grade of hypercoagulability had significantly higher number of metastatic sites (4 and more vs. 1–3; P = .02).

### Association of hypercoagulability with disease-progression under immunotherapy. A case-control study

Two groups of patients were compared in a study. Baseline characteristics were well balanced and these groups were compared by modified MSKCC prognostic score including predictors of short survival from ARCC trial (Table 3).

**Table 3:** Study and control groups.

	**Study group**	**Control group**	Differences between groups, *P value*
hypercoagulability	+	-	-

number of patients	28	28	-

male/female	20/8	21/7	0.33

median age	62	60.1	0.52

**Prognostic factors**			

Good prognosis	15 pts (53.6%)	15 pts (53.6%)	-

Poor prognosis	13 pts (46.4%)	13 pts (46.4%)	-

Sixteen patients of study group (57.1%) and eight patients of control group (28.5%) had disease progression after 2 treatment cycles. Differences between two groups were significant (P = .003).

Disease control rate (Complete response (CR) + Partial response (PR) + Stable disease (SD) was significant higher in patients with normal coagulation: 1 (3.6%) CR + 5 (17.9%) PR + 14 (50%) versus 0 CR + 1 (3.6%) PR + 11 (39.3%) SD (P = .003).

**Figure 1:**
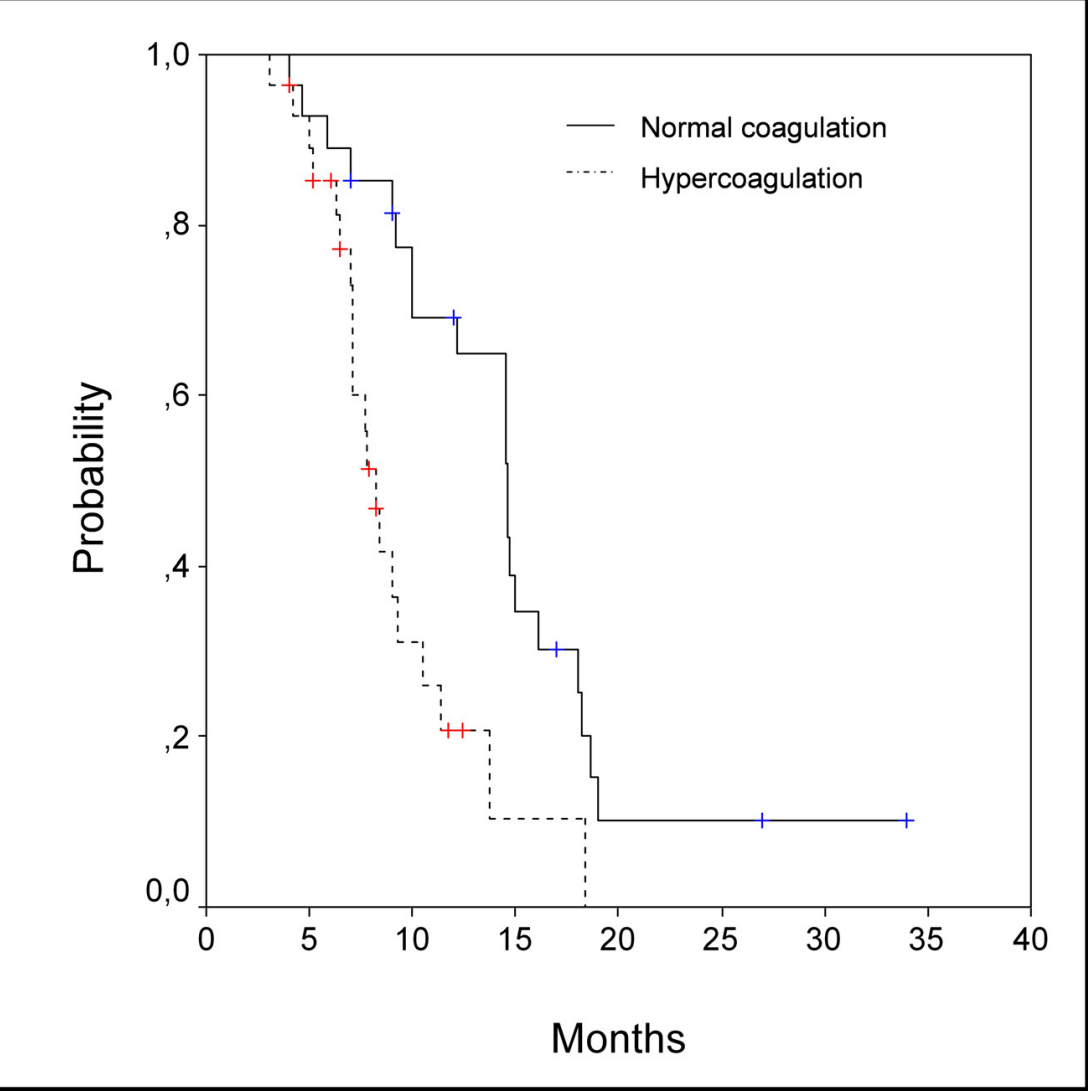
**Overall survival (Kaplan-Meier analysis)**. Median overall survival was 8.2 months for group with hypercoagulability, and 14.6 months for group with normal coagulation. Differences were significant (HR = .54, P = .0011).

In Kaplan-Meier analysis, patients with hypercoagulability had a significantly shorter overall survival than patients with normal coagulation. Median survival was 8.2 (95%CI 7.2–9.2) and 14.6 (95%CI 12.4–16.8) months, respectively (HR = .54, P = .0011). Survival curves are given in Figure 1.

### Multivariate analysis

In univariate analysis, patients (N = 289) with hypercoagulability had significantly shorter survival than patients with normal coagulation; median survivals of 8.9 and 16.3, respectively (P = .001).

Additional factors that were also associated with poor survival were MSKCC prognostic factors, increasing ECOG performance status, shot time from diagnosis, non-clear cell histology, and the presence of liver or bone metastasis, more than 1 metastatic site.

Because of the large number of factors that were associated with hypercoagulability and/or survival in general study population, multivariable analyses were conducted to determine whether hypercoagulability was an independent predictor. The results of this analysis are summarized in Table 4.

**Table 4:** Multivariate analysis

**Factor**	**Estimate + SE**	**Hazard ratio (95% CI)**	**P**
MSKCC risk group	0.56 ± 0.07	1.75 (1.65; 1.85)	< .001

Hypercoagulability	0.51 ± 0.09	1.63 (1.5; 1.76)	< .001

Non-clear RCC	0.29 ± 0.10	1.35 (1.21; 1.49)	.002

≥ 2 metastatic sites	0.27 ± 0.09	1.3 (1.1; 1.5)	.003

Age > 60 y	0.25 ± 0.08	1.26 (1.05; 1.47)	.007

SE – standard error; CI – confidence interval, P – P value (Wald test)

By using stepwise variable selection, hypercoagulability, MSKCC risk group, non-clear RCC, number of metastatic sites, and age were found to be independent predictors of survival.

## Discussion

Although advances in the treatment of metastatic RCC have been made in recent years, the overall outcome of this disease remains dismal. Despite encouraging results with new treatment agents, their optimal incorporation into clinical practice remains to be defined. Whether these agents should be used as monotherapy or combined with cytokines or other agents remains speculative. The role of prognostic factors may help to define better these questions.

We sought to analyze metastatic RCC patients before cancer-specific treatment in N.N. Blokhin Russian Cancer Research Center. The objective of this study was to determine whether an elevated coagulation level is a negative predictor for survival and response to treatment in metastatic RCC. Coagulation estimate is a simple, inexpensive test that can be obtained before treatment and could help to individualize therapy based on risk factor assessment. Our results showed that 40% of patients had hypercoagulability at treatment start. Hypercoagulability can be an independent prognostic factor according to our data.

There were no studies which demonstrated prognostic role of hypercoagulability and impact on response to immunotherapy in metastatic RCC patients. However, influence of disorders in the cellular hemostasis on survival of RCC patients was shown.

In the retrospective study by R. Suppiah et al. [9], 192 of 714 (25%) metastatic RCC patients had thrombocytosis. In univariate analysis, patients with thrombocytosis had significantly shorter survival than patients with normal platelet count. Median survival was 8.4 months and 14.6 months, respectively (P < .001).

In another retrospective review by Symbas et al. [10], 147 of 259 (57%) metastatic RCC patients were found to have at least once platelet count of > 400,000/μL before treatment. Mean survival for these patients was 92 months, compared with 151 months for those with normal platelet count (P = .005). Conclusions from this study were that thrombocytosis could be manifestation of aggressive tumors, with worse survival when compared with patients with normal platelet count.

In a French study with more than 700 patients treated in multicenter trials of cytokines, thrombocytosis was found to be a significant predictor for survival on univariate analysis [11].

The exact mechanism causing hypercoagulability as well as thrombocytosis in association with RCC is unclear. Possible mechanisms include overproduction of tumor procoagulant and cytokines/growth factors stimulating tissue factor pathway and megakaryocytes in case of thrombocytosis.

Tissue factor is a glycoprotein responsible for initiating extrinsic pathway of coagulation. Immunohistochemical studies show that renal cancer cells express tissue factor on their cell surfaces. Also, tissue factor antigen was detected in the endothelium of vascular channels within the renal tumors [12].

In vitro experimental studies demonstrate that interleukins (IL), such as IL-6, IL-1 are able to cause hypercoagulability through stimulation of tissue factor activity [13-15]. More than half of patients with metastatic RCC have increased levels of circulating IL-6, which also correlates with increased C-reactive protein levels. In a study by Walther et al. [16], IL-6 was detected in 19 of 21 (90%) renal cancer cell lines obtained from 20 patients wit metastatic RCC and also detected in the serum of 33 of 59 (56%) patients with metastatic RCC. Elevation of the cytokines was associated with paraneoplastic manifestations including coagulation disorders.

Several theories have been proposed on how hypercoagulability plays a significant role in tumor growth. One way is an impact on proliferation and metastasis. The studies of fibrinogen-deficient mice directly demonstrate that fibrin(ogen) plays an important role in cancer pathophysiology and is a determinant of metastatic potential. Fibrin(ogen) appears to facilitate metastasis by enhancing the sustained adherence and survival of individual tumor cell emboli in the vasculature of target organs. Fibrin degradation products have been reported to have angiogenic, chemoattractant, and anti-inflammatory activities and these proteolytic derivatives of fibrin might also be of biologic relevance to tumor progression. Thrombin induces proliferation of metastatic cells [17,18]. Influence on angiogenesis is the second important tumor growth mechanism of hypercoagulability. Tissue factor and thrombin are two substances which stimulate angiogenesis directly [19-21]. Conversely, tissue factor and factor VIIa inhibitors, as well as antithrombin block angiogenesis and tumor growth [22,23].

Thrombi clots contain a variety of factors such as vascular endothelial growth factor (VEGF), fibroblast growth factor (FGF), platelet-derived growth factor (PDGF), transforming growth factor beta (TGF-β), IL-6, thrombin, and fibrinogen, platelets. These modulators have been implicated in various steps of tumor progression and in the development of metastases [24]. A positive correlation between serum VEGF levels and disease progression was discovered in patients with different advanced cancers [25]. Being one of the most significant proangiogenic cytokines, FGF contributes to migration, proliferation, and differentiation of endothelium cells, and regulation of the expression of proangiogenic molecules [26]. PDGF induces angiogenesis by means of stimulation of VEGF expression in tumor endothelial cells and by recruiting pericytes to new blood vessels [27]. TGF-β plays an active role in platelet aggregation and regulation of megakaryocytes activity. This cytokine also regulates the activity of the VEGF system and enhances endothelial cell survival [28,29]. Stimulation of growth factors and expression of their receptors by thrombin and tissue factors has been detected in many trials [21,30,31].

## Conclusion

Our study confirms the prevalence of hypercoagulability associated with metastatic RCC. We have also demonstrated that hypercoagulability determines worse survival and response to treatment for metastatic RCC. With further studies, this single independent prognostic factor may provide a simple approach to improved risk stratification of patients in future clinical trials protocols.

## Competing interests

The authors declare that they have no competing interests.

## Authors' contributions

IT, LV participated in the design of the study and performed the statistical analysis as well as drafted the manuscript. AM, OS, AY carried out the laboratory studies, participated in the interpretation of the laboratory data. IT collected patient's data. All authors read and approved the final manuscript.
